# Changing the Home Food Environment: Parents’ Perspectives Four Years after Starting Obesity Treatment for Their Preschool Aged Child

**DOI:** 10.3390/ijerph182111293

**Published:** 2021-10-27

**Authors:** Paulina Nowicka, Johan Keres, Anna Ek, Karin Nordin, Pernilla Sandvik

**Affiliations:** 1 Department of Food Studies Nutrition and Dietetics, Uppsala University, 752 37 Uppsala, Sweden; paulina.nowicka@ikv.uu.se; 2 Division of Pediatrics, Department of Clinical Science, Intervention and Technology, Karolinska Institute, 141 57 Huddinge, Sweden; anna.ek@ki.se (A.E.); Karin.nordin@ki.se (K.N.); 3 Dietitian Unit, Region Sörmland, 611 88 Nyköping, Sweden; Johan.Johansson.Keres@regionsormland.se

**Keywords:** covert control, overt control, child development, food environment, obesity

## Abstract

Changing the home food environment is key to childhood obesity treatment. However, new challenges arise as the child grows older. This study investigates parents’ views on the longer-term management of the home food environment, 4 years after starting obesity treatment for their preschool-aged child. Semi-structured interviews were conducted with 33 parents (85% mothers, 48% with a university degree) of 33 children (mean age 9.3 (SD 0.7), 46% girls) from Sweden. The interviews were analyzed using thematic analysis. Two main themes were developed. *Making changes in the home food environment* illustrates the types of changes families make over time in relation to child development. It consists of three subthemes: covert changes, overt changes and child-directed changes. The second theme, *an ongoing negotiation*, captures parents’ experiences of managing the home food environment as a continuous process of balancing and recalibrating in relation to present challenges and concerns about the future. It includes three subthemes: concern and care, two steps forward one back and maintaining everyday balance. Managing the home food environment is a constant process affected by everyday life, parents’ strategies and the child’s development. Our findings can strengthen childhood obesity treatment and help prepare parents for challenges that lie ahead.

## 1. Introduction

The prevalence of overweight and obesity among children has increased globally over the past 20 years [1]. Obesity is classified as a chronic disease and it is of great importance that children receive treatment [2,3]. While obesity treatment for young children is rare, treatment at an early age seems to have the most beneficial effect [4,5], and interventions have shown promising results with clinically significant weight loss [4] and a lower energy intake up to 1 year after starting treatment [6].

One key factor in childhood obesity treatment is the management of the home food environment [7]. This consists of various factors which interact and affect the child’s eating habits [7,8], including access to food, the meal environment and parents’ actions around the meals, such as planning, cooking and eating [8,9]. Rosenkranz and Dzewaltowski [7] constructed an ecologically informed model of the home food environment relevant to childhood obesity with three domains. The first domain is the “built and natural environment” and includes, for example, availability and accessibility of foods at home, tablescapes and cooking equipment. The second domain, “political and economic environments”, includes socioeconomic status and family food insecurity. The third domain, “sociocultural environments”, includes customs and traditions, family structure, stress and schedules, rules, family eating patterns, nutritional knowledge and food preparation skills. According to this model, the home food environment develops through an interaction between parents, children and the wider environment in which they live [7]. Parents influence the home food environment directly through purchasing and preparing food. They also influence the home food environment indirectly by setting rules about food and eating, acting as role models and speaking about food with the child. The child, in turn, influences the home food environment through their food preferences and behaviors related to food, such as asking for food in general or for specific dishes. In the first years of life, parents have a great influence on their child’s eating habits [10]. As the child grows older, the parents’ influence over the child’s food intake decreases because children’s environments become more diverse, expanding beyond the parents’ remit [11].

Changes to the home food environment can be difficult to implement [12,13,14] and require parents’ commitment to the process of change. Previous studies have explored changes parents make in the home food environment through the five-step behavior change model [15,16]. According to this model, parents move from being unaware of the problem and having no intention of changing their behavior to acknowledging the problem, planning for change, modifying the behavior and finally maintaining the new behavior to the extent that it is incorporated into their lives [15]. Using this model, researchers have found that parents who have a higher level of engagement progress further in the process of changing their child’s eating habits [16]. However, the process of changing the home food environment is multifaceted, and parents employ different strategies to achieve positive changes. One study found that parents implemented rules and changes regarding eating in the family as a whole in order to maintain a child’s healthy eating [12], while a Swedish study found parents of preschoolers involved them in making changes [14]. A qualitative study from the USA found that parents of children aged 6 to 12 years planned meals, cooked simple dishes, offered alternatives when the child requested fast food or let the child have fast food on fewer occasions [13].

Strategies used by parents to develop a healthier home food environment can be categorized as overt or covert control [17]. Overt control means that the child can notice the strategies the parent uses, for example, when the parent encourages the child to eat more or less of particular foods. Overt control can contribute to both healthier and less healthy eating habits, depending on how it is performed [18]. Covert control is more difficult for the child to notice, and may include, among other things, avoiding purchasing food that the parent does not want the child to eat [17]. Previous research has found mixed results regarding the association between covert control and children’s weight status. In two cross-sectional studies no association was found [17,19], while a prospective study showed an association with lower weight status [20]. Of note, a longitudinal study found a link between mothers’ covert control and healthier eating among preschool age children [21], suggesting that longitudinal research may be needed to appreciate the effects of covert control on weight outcomes.

Research shows that obesity treatments for children have a significant effect on children’s weight status [4,5]. However, while obesity is a chronic condition that needs ongoing management, it is unclear how treatment programs may support families in maintaining the intervention effects, including keeping a healthy home food environment as the child grows older. This study aims to investigate parents’ views on the longer-term management of the home food environment, 4 years after starting obesity treatment for their preschool-aged child.

## 2. Materials and Methods

This study is based on semi-structured interviews with 33 parents who were enrolled in the More and Less (ML) study for 12 months. The ML study was a randomized controlled trial with 177 participating families [22]. Families with children aged 4 to 6 years diagnosed with obesity were recruited to the trial between 2012 and 2016; families were included in the study only if the child did not have additional health or developmental issues that could influence weight status and if the parents were able to communicate in Swedish. Half of the families were randomized into a group that was offered standard treatment in outpatient pediatric clinics in the Stockholm Region focusing on behavior changes around diet and physical activity. The other half of the families were randomized into two groups; both groups received the newly developed ML parental support program, but only one received additional booster phone calls in four-to-six-week intervals after the program ended [23]. The program consisted of weekly 90 min meetings over 10 weeks, where only the parents participated. The program focused on evidence-based, positive parenting strategies with themes such as how to be a good role model, how to encourage your child, how to create rules and routines, how to set limits and handle power struggles with your child and how to handle stress and ask the child’s network for support.

The primary outcome was a mean change in the children’s weight status, body mass index z-score (BMI z-score), 1 year after the start of treatment. The results showed that the group receiving the ML program with follow-up phone calls had a greater reduction in BMI z-score compared with the group not receiving follow-up phone calls and compared with standard treatment [22]. In the present study, overall parental views on the longer-term management of the home food environment, regardless of the treatment they received during the ML study, were explored. The study was approved by the Regional Ethical Board in Stockholm (ID: 2011/1329-31/4) with amendment (ID: 2016/80-32). Informed consent was obtained from all participants.

Families who were recruited in the first 2 years of the ML study were invited to take part in this 4-year follow-up. Of the 67 families we approached, 33 agreed to participate (18 declined, 14 were unreachable and 2 had moved abroad). Of the 33 parents interviewed, 28 were mothers and 5 were fathers. Seventeen of the families participated in standard treatment and 16 in the ML program. More information about the participants can be found in Table 1. Compared to families who were not interviewed, parents in the included families had a higher education level, and fathers were more likely to be classified as having a lower weight status. Telephone interviews were conducted by an experienced pediatric nurse, a member of the ML research team. The average interview time was 45 min, ranging from 28 to 70 min. The interview guide addressed the following topics: what parents found helpful about the treatment they had received, how families maintained new habits, how children perceived their bodies, what parents found easy or difficult to change, who in the child’s network had been involved in the child’s weight management, how parents spoke to their children about weight-related issues, how parents viewed their parenting and how treatment had affected the family.

The interviews were transcribed verbatim and the transcript extracts focusing on the home food environment at the microlevel were selected for analysis, based on the model of the home food environment pertaining to childhood obesity [7].

The interviews were analyzed (initially by J.K.) using thematic analysis [24]. The texts were first condensed and divided into meaning bearing units. This became the starting point for the initial coding. After further analysis, each condensed text unit resulted in one or several codes that carried across text units with similar content. Subsequently, all codes were compiled and discussed jointly among J.K., P.S. and P.N. Throughout the analysis process, codes and categories were discussed and adjusted by J.K., P.S. and P.N. The process resulted in two main themes, each comprising three subthemes. Quotes from mothers and fathers that exemplify the thematic categories and subthemes are provided. Participant code names denote the relation to the child (M = mother, F = father). The number after M and F is the interview order code.

## 3. Results

**Figure 1:**
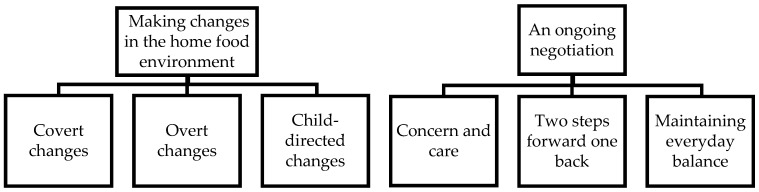
Main themes and subthemes.

Two main themes were developed: “making changes in the home food environment” and “an ongoing negotiation”. These two themes and respective subthemes are illustrated in Figure 1.

### 3.1. Making Changes in the Home Food Environment

The first theme illustrates the types of changes in the home food environment that families made over time in relation to child development. The changes varied between the families; however, parents commonly reported exchanging energy dense nutrient poor (EDNP) foods with healthier options, reducing portion sizes or limiting the intake of foods such as sweets, snacks, ice cream, cookies, buns and soft drinks. This theme is divided into three subthemes, covert changes, overt changes, and child-directed changes.

#### 3.1.1. Covert Changes

Parents perceived replacing EDNP foods with healthier alternatives as the easiest change to implement. They described reading the ingredient list and nutrient information on the back of food packages, looking for food labels such as the Keyhole (a label that designates the healthiest options within a specific food category, used in Nordic countries) and purchasing more vegetables. One mother said: “I guess you should think about buying a little more according to the plate model when shopping for food. That was easy” (M12). The plate model is a pedagogical tool developed by the Swedish Food Agency, illustrating how food can be distributed on the plate to increase the amount of vegetables and get a good balance of energy and nutrients in the meal. Parents also said they reduced the availability of EDNP foods and beverages, such as soft drinks, juices, ketchup, sugary cereals and chocolate milk. One parent describes it like this: “These choices, like, they almost always drank chocolate milk, for example, now we do not even have it at home” (M5).

Changes in food purchasing were linked to changes in food preparation, and parents said they started to cook more food from scratch and tried to avoid processed foods. One mother, for example, described buying an ice cream machine for the summer to prepare homemade ice cream with a lower energy content. With greater awareness about which ingredients contributed to a home-cooked dish’s energy density, parents also adjusted their cooking. A father described how he implemented changes in cooking: “So it is more *us*, our behavior as parents that has changed, that we know more what is appropriate, that you should not have so much oil and butter in the food and so on” (F56).

Across the sample, EDNP foods were not brought home routinely, but only for special occasions. Some parents limited EDNP foods to parties or other festivities, as one mother described it: “So in the end we just set the limit that he could only eat such things [EDNP-foods] when it was a party, not even, not on weekends just because it tastes good. Only at parties and on your birthday” (M12). Others kept serving EDNP foods as part of *Cozy Friday* and *Saturday sweets* and instead limited the amount eaten. Cozy Friday and Saturday sweets are weekend-specific practices embedded in Swedish culture [26]. Cozy Friday often involves enjoying the evening together as a family, having dinner and watching TV shows while eating snacks such as chips. Saturday sweets means that children are allowed to buy some of their favorite sweets and eat them on that day, providing a structure in which sweets can be a part of a child’s diet in an acceptable and regulated way (ibid). One mother described how her partner opted to replace all snacks with healthier options on Cozy Friday, while she sought a more balanced approach:

When it comes to chips, for example, he [dad] seemed to like switching entirely to fruits and berries and nuts and so on instead of having chips on Fridays… or sweets. But I felt that you cannot ban [unhealthy snacks] completely—they are children.

(M83)

#### 3.1.2. Overt Changes

After treatment, parents felt better able to motivate and support the child in having smaller portions and limit snacking. However, parents said it was difficult to deny their hungry child a second portion of food, especially if this could lead to arguments at the dining table. Therefore, they used overt strategies to limit the children’s food portions while avoiding conflicts. For example, parents cooked enough food only for a first portion, did not put the pots on the dinner table, divided the portion into two smaller servings, used smaller plates, reminded the children to take one portion or let the children serve themselves with a meal measure (a measure used to estimate volumes—one for vegetables, one for carbohydrates and one for protein designed according to the plate model). The parents adjusted and adapted their strategies over time to respond to the children’s needs, as one mother described:

Because sometimes when you give him one portion then he says “no I want more I want more!”… Then I have asked what I should do. “Well, serve him smaller portions, but serve him twice.” In the child’s mind, it’s like a habit to always have two servings.

(M54)

Parents also described trying to find alternatives to Saturday Sweets and Cozy Friday such as more festive dishes, vegetable sticks with dip or fruit salad, or non-food-related items such as a small toy or a magazine. Replacing EDNP foods with healthier alternatives was perceived as easier than just saying no:

… yes, that’s probably what’s most difficult…, having to say no to an ice cream or…, we have tried to find like, she might get to buy a magazine instead or something like that in the store. Because before it was a Kinder egg (a chocolate egg with a small toy inside) or it was an ice cream.

(M6)

While parents sought healthier alternatives, they also emphasized it was important that children continued to enjoy sweets and did not start to associate sweets with feelings of guilt. One strategy was to involve the child in choosing sweets or snacks:

…then you have to choose if you want popcorn or if you want something else or if you want sweets and then we stick to our hundred grams, that’s what you get and they get to choose them [the sweets], pick what they want and that’s what it is, there’s nothing more…

(M14)

Despite parents’ strategies, some children challenged the food rules and limits the parents had created. For example, one mother describes how angry her daughter became when asked to wait for food:

But she can get angry now and then, and I understand that she may be hungry, but I usually say that “you have to wait a while”. “Now we wait a bit and see, we can do this instead”, but sometimes she does not want to wait, sometimes she just gets really angry and maybe throws some pens or something like that, you know, and just aaahhh! Or walks away and slams the door. But it blows over pretty fast, but she can, she can definitely get quite angry if I say no.

(M24)

In some cases, it seemed these challenges stemmed from the parents’ rules being unclear or inconsistent. When parents made overt changes there was more room for the child to be creative in getting around the rules. For example, children could serve themselves a second portion of food when the parents were not looking, or sneak into the kitchen and grab something to eat.

… if he serves himself food. If you do not notice that he serves himself more and he like… you could say sneaks, although it is a little difficult to sneak at a dining table… But he openly takes another serving if neither of us react.

(F96)

#### 3.1.3. Child-Directed Changes

Parents described individual differences in how children accepted changes in the home food environment. Some stated that their children collaborated with them from the beginning of treatment, others said their children initially resisted changes, but this resistance passed quickly, and still others said it took some time. One mother describes how her child instantly accepted changes in the home food environment:

It was the same thing when I started talking to her “well, now we will do this. Okay?” … she has made it super easy for us. But then that has also made it easier, like, when there is no nagging from her all the time, then it has been much easier to keep it [the new habit].

(M5)

Several parents described their children as motivated and committed to changing the home food environment, saying their children became more aware of healthy eating habits and came up with their own suggestions for alternatives to sweets or snacks. For example, parents mentioned that their children helped to apportion their sweets, reminded the parents not to eat buns and cookies on weekdays, participated in menu planning and chose Keyhole-labeled foods in the store. A mother described how her daughter reminded her to choose healthier options when shopping for food:

Sometimes you forget as an adult and just grab whatever you are used to. Then [name of daughter] can say “mom [name of dietician] said we should not take that one”.

(M6)

As the children grew older, parents experienced new challenges. These required new approaches to motivate children and to make positive changes. For example, with age, children became more independent and stayed alone at home for longer periods of time. Some parents discovered their child started snacking or eating large amounts of foods and unhealthy snacks when they got home from school. Parents felt that their influence over their child’s eating decreased over time and tried to find new strategies. A mother described adjusting which foods were available at home:

Yes, but you have to think about that as well, what [type of food] you have at home and were you keep it, because I cannot control her all the time… And that, she says that herself “If I see it, it’s so hard to resist”. Which is insightful of her, I think. And then, after all, my mission is simply to make sure that it is out of sight.

(M85)

Over time, parents explained their food rules to their children, saying that when children understood, they found it easier to accept the rules. The task of explaining became less challenging as children grew up:

Now he understands things in a completely different way. He’s a little older now too. He is eight now, it is possible to communicate with him. He understands the connection. So, he did not understand at first. But nowadays it’s a little easier to work with him.

(M86)

Older children needed to take greater responsibility for their own eating and weight management. Parents perceived this shift in responsibility as a challenge, but also as an opportunity to support their child’s growing independence and enable them to make healthy choices:

It’s like the older they get, the harder it is, or maybe not harder, rather it is not possible to control in the same way. I feel that I as a parent need to give her the responsibility of how much she eats and what she does not eat and so on. When she was five years old then it was like “yes” or “no”, now it’s more “if you eat this maybe you cannot eat that…”. Today I have to help them by giving alternatives instead. That she gets, that you help her making the decisions instead of making the decisions for her, and that is a challenge in its own way.

(M58)

### 3.2. An Ongoing Negotiation

This theme captures parents’ experiences of managing the home food environment as a continuous process of balancing and recalibrating, in relation both to present challenges and concerns about the future. It includes three subthemes: “concern and care”, “two steps forward, one back” and “maintaining everyday balance”.

#### 3.2.1. Concern and Care

Parents’ main concern was that changing the home food environment might have unforeseen consequences, such as disordered eating, when their child was older. Although parents said they focused on health rather than weight when discussing healthy eating, they were concerned that children might become self-conscious about their weight. Some parents were also concerned that restricting EDNP foods would eventually lead to overconsumption of these foods:

… when you have forbidden something. That may be why what is forbidden becomes what you want to eat later, when you have the opportunity to buy it yourself. Do you know what I mean? When they are old enough, that [the restrictions] affects them negatively instead? So, I don’t know, but that’s what time will tell us. You just hope that you are do the right thing.

(M88)

Other parents felt confident about the changes they had made to the home food environment. This feeling was strengthened when the changes became an established habit and the child’s weight had stabilized:

We do not have to worry about anything, about his weight, or how he grows or anything like that, I can at least feel that I don’t have to keep the same focus on it. …The routines around the portions and so on is in the back of my mind.

(M70)

Some parents worried that they were giving their child too little food and that the restrictions may contribute to the child not getting enough nutrients, as one father explained: “Sometimes we are even afraid that we give him too little food. So, we are afraid that he will be malnourished sometimes …” (F56). Parents also said they felt sorry for the child when they restricted their food intake, finding it particularly difficult when the child was hungry and asked for a second serving. A mother described how she experienced these situations:

It is very difficult sometimes … when she says that she is hungry and she has not been able to eat at school, you get a bad conscience, you do not want her to be hungry.

(M6)

Other parents reported feelings of guilt because they had not implemented sufficient changes or had not maintained the changes over time. In these cases, parents expressed concern for their child’s health and well-being. In the parents’ stories, they suggested that long-term health was sometimes opposed to short-term well-being. To balance between short-term and long-term concerns, parents felt they needed to be careful when restricting portion sizes or EDNP foods, adapting the restrictions to the current needs of the child. A father described how he gradually limited sweets, snacks and ice cream so that the child would become used to these changes without being distressed:

They have almost stopped asking for candy; first they said “Saturday sweets”, no that did not happen, then we had trail mix, that happened a few times, but that is over now… Regarding ice cream, … they get it every now and then, but not that often. “Dad, I haven’t eaten ice cream in a week.” “Well, that’s great, that is really, really good”, I answer and [they say] “no dad we have to have it”, but that does not help much. I try to do things step by step.

(F33)

Although the parents said they became better at supporting their children around eating habits, some were concerned about how their child will eventually manage their food intake on their own. As one mother explained:

… because she is so old now and what is my biggest concern, or what has already started, is that she eats when I am not around. That’s why she has gained so much weight this year. She eats snacks, when she comes home from [unclear] and she can buy something to eat herself and so on.

(M67)

#### 3.2.2. Two Steps Forward, One Back

Parents described the work of change as a process that required ongoing focus and motivation for both parents and children. One mother said it was easy to fall back into old habits:

Because we treat ourselves now and then, we notice that we can fall into old habits for a period too, but then I feel that “no this is not a god thing, now we, like, have to get back to our good routines”.

(M58)

What changes that became a new habit varied between families. Some parents believed it was easy to maintain restrictions on EDNP foods, while others stated that limiting portion sizes was easier to uphold. Over time, parents noted that, the children began to eat larger portions, increased their intake of EDNP food, or gained weight. This required taking new steps toward behavior change, which could be seen as tedious and time-consuming. Parents said the knowledge they gained through treatment was still useful, and those who still had contact with a healthcare professional said that planned visits increased their motivation to maintain implemented changes.

Some parents described how, over time, their children began to challenge new rules and routines, requiring new compromises. For example, when a child asked for a second portion of food or demanded sweets in the middle of the week, parents sometimes bent the rules and provided them with the food they requested. According to parents, compromises took individual circumstances into account, e.g., the child being more physically active, or the parent’s own emotional capacity, being unable to say no and face conflict on that day.

Parents were aware that changes in the food environment needed to be maintained over time, but that this required a long-term commitment, which one mother described as follows: “… it is a lifestyle change, it is not like a course and then you are done, we will need to continue for the rest of our lives” (M21). Even if some new habits were established, others changed, and it was easy to fall back into old behaviors.

#### 3.2.3. Maintaining Everyday Balance

Parents’ ability to manage the home food environment varied from day to day. A stressful day could mean that parents did not have enough time to cook or did not have the energy to negotiate their children’s demands for extra portions or EDNP food. For example, some parents said that, when they were ill, they lost their energy to cook good food, help the child to limit their portions or support the child when they said they were hungry. However, when they recovered and gained more energy, they could resume managing the home food environment. Nonetheless, managing the home food environment was challenging not only in exceptional circumstances of a parent’s illness, but also in the everyday flows of a busy family and work life, which parents felt was an obstacle in the work of change. A hectic workday, this mother explained, affected the energy she had to support her child:

Well, what can be difficult, for example, when you have worked and are tired, something may have happened at work, and then it may be that you do not have the strength. “Okay do what you want!”… It has not been easy all the time.

(M54)

Lack of time and energy made parents less consistent in applying changes to the home food environment. This, the parents suggested, contributed to the child starting to challenge food rules, which could easily turn into conflicts. To overcome such inconsistencies, some parents set up routines they could follow every day; for example, this mother explained she planned meals to maintain consistency:

But it is super important to dare to stay put, to have the guts to say no and stick to it [your rules] and not start negotiating or that the child starts negotiating. It will not be good. Then I think that planning is better, just to plan the meals in advance, … to have a plan before a sharp situation arise. To be prepared at all times—then you can be calm and nice and harmonious.

(M59)

Parents said that being consistent and clear with set rules and routines made everyday life easier. This involved practices such as cooking every day, meal planning, buying ready-made lunch bags or preparing dishes the day before. However, to maintain these practices, some parents had to make wider life changes. One mother said that she reduced her working hours to be able to have food on the table when the child came home at five o’clock.

Everyday routines were difficult to maintain during longer holidays or vacations, when the intake of EDNP foods increased and mealtimes changed. Some parents also said it was easy to revert to previous routines, such as having coffee with buns or cookies, a habit which they had eliminated after treatment but resumed on holidays. When holidays ended, parents restored the home food environment but felt that it took some time before their family could resume healthier eating habits.

## 4. Discussion

This study examined parents’ views on how they managed the home food environment 4 years after starting obesity treatment for their preschool aged child. Parents described changing the home food environment as an ongoing process. Some parents made several changes at once, while others made one change at a time. However, regardless of the pace of change, the home food environment was transformed through a number of small changes that differed from family to family. The process of changing the home food environment was not described as linear, however. Parents moved back and forth between the stages of change, and it was easy to fall back into old behaviors. Some parents said they resumed old behaviors because of their limited capacity to maintain changes in the food environment, while others said they did so to meet their child’s needs and wishes. Decisions about what was eaten and how often were made in negotiation between parents and children. Previous studies have shown that adolescents follow their parents’ behavior regarding good eating habits, and several parents also changed their own eating habits to set a good example [27,28].

Parents creatively adapted the dietary advices provided during treatment to develop practical solutions in their home food environment. A key change was reducing the availability of unhealthy foods in the home and increasing the availability of healthy foods. Previous studies have shown that such changes contribute to an increased intake of fruits and vegetables [29] and a reduced intake of EDNP foods [30]. Several parents said they learned to make better choices in cooking and eating, such as reducing the use of fatty ingredients, using Keyhole-labeled products and increasing the proportion of vegetables. This is an important aspect of lifestyle changes, as parents’ food knowledge is associated with their children’s healthy diets [7]. Moreover, changes in food choices together with restrictions of the intake of EDNP food seem to be in line with recommended dietary advice in Sweden [31]. Most families had implemented many changes already from the start of the obesity treatment [32], and kept these changes over the 4 years.

When making changes in the home food environment, parents were sensitive to children’s will and needs, adjusting their practices accordingly. A previous study has shown that parents who perceived their child as heavier were more likely to use covert control [17]. The parents in this clinical sample used both overt and covert control in their efforts to influence the home food environment. They felt that covert control, such as avoiding having EDNP foods at home or exchanging foods with similar but healthier alternatives, was easier to perform. It has been described previously that parents are reluctant to use overt control to avoid conflicts at the dining table [21]. The parents in our study partly shared that view. To avoid conflicts when limiting a child’s food portions, as the child became older, the parents involved the child in a dialogue about rules and routines and healthy foods, giving the child more control.

Parents further displayed a complex understanding of children’s health, which could not be reduced to weight status alone. They described seeking balance between short-term and long-term wellbeing, as exemplified in descriptions of allowing children to take part in Swedish food traditions, such as Cozy Friday or Saturday sweets, while limiting their intake of treats or exchanging some EDNP foods with healthier alternatives. A similar understanding of children’s wellbeing and the need for balance was described in the Oregon-based Grandparents Study, in which parents and grandparents of preschoolers explained that it was important for children to receive treats, particularly in the context of making memories and bonding with their grandparents [33]. Additionally, parents sought a balance between everyday routines and special occasions, such as holidays, when food rules were often bent. However, parents found that returning to healthier eating routines after holidays was a slow process, a finding that aligned with research that found temporary deviations from routines make it difficult to maintain healthy eating habits [34].

Maintaining changes was difficult when faced with the uncertainties, busyness, and stress of everyday life. Parents who experienced sick leave also said their ability to cook or support their child at meals was greatly reduced when they were ill. Previous studies have found that parents’ hectic everyday life is a main obstacle to children’s healthier diets [12,13]. Furthermore, in households where parents experience higher levels of stress, there is lower access to healthy foods [35]. Maintaining changes was also difficult when children challenged food rules and routines. To overcome this, parents talked to their children about healthy eating habits and included them in making changes. This is aligned with a previous study highlighting the importance of cooperation between children and parents in achieving a healthy diet [13] and with another study that found that involving the whole family in lifestyle changes is key to consolidating new eating habits [12]. Additionally, as children grew older, they became more independent eaters. This posed some risk to healthy eating habits, as young people’s intake of healthy foods decreases when they share the food environment with their families to a lesser extent [11]. To regulate older children’s eating, parents focused on the availability of certain foods at home. A previous study among teenagers showed that parents continue to have a great influence on the child’s diet by limiting or increasing the availability of various foods in the home [27].

This study has a few limitations. While childhood obesity is more common in families with lower education [36], parents with a university education were overrepresented in the sample, and their experiences of the home food environment might not fully capture parents’ experiences across socioeconomic backgrounds. In addition, like other studies in the field, fathers were underrepresented in this study [37]. As mothers and fathers respond to childhood obesity treatment differently [38], we suggest that future studies should include both parents. However, as most of the mothers we interviewed said they had the main responsibility for food changes in the home, the lower number of fathers in this study did not detract from the findings.

The findings have several clinical implications. Four years after the start of childhood obesity treatment, parents still perceived the knowledge they gained during the treatment as useful, and those who continued to have treatment felt that visits with healthcare professionals increased their motivation to maintain lifestyle changes. However, it is important for healthcare professionals to understand that changes in the home food environment can take time and that individualized solutions are needed to address families’ needs as children grow older. Treatments would also benefit from a strong focus on strategies and tips for handling challenges that may arise in life, such as the growing child’s refusal to follow food rules, stressful periods at work, a parent’s illness, holidays or travelling. In addition, long-term healthcare support would enable parents to maintain positive changes in the home food environment over time.

## 5. Conclusions

The knowledge parents gained through childhood obesity treatment enabled them to make positive changes in the home food environment. In the 4 years since starting treatment, parents responsively adapted these changes to the child’s age-related needs, using both covert and overt control. The process of changing the home food environment was not a straight path forward, and it could be difficult to establish new habits, particularly when faced with new stresses in everyday life and a growing child who challenges food rules; however, parents made an ongoing effort to maintain a healthy food environment in the home. In maintaining a healthy home food environment, cooperating with the child was key, and parents sought to include children in food decisions. Moreover, parents attempted to balance concerns about children’s long-term health with short-term emotional and social wellbeing. As such, rules and routines were occasionally relaxed—for example, during holidays—so as to allow children to participate in food traditions and receive treats, while not abandoning the larger process of establishing healthier eating habits. All considered, parent-maintained changes in the home food environment is an ongoing process, and they must adapt it creatively and flexibly. These findings suggest that childhood obesity treatments should address how to handle challenges that arise as children grow older, as well as provide parents with longer-term support.

## Figures and Tables

**Table 1:** Descriptive statistics of the parents and their children.

Treatment, n (%)	
More and Less parent support program	16 (49)
Standard treatment	17 (51)
Parents	
Age (years), mean (SD)	41.1 (5.6)
BMI (kg/m^2^), mean (SD)	28.6 (5.6)
Gender, woman, n (%)	28 (84.8)
Foreign background *, n (%)	14 (45.2)
Weight status **, n (%)	
Normal weight	12 (38.7)
Overweight	10 (32.3)
Obesity	9 (29)
Missing values	2
Highest completed education, n (%)	
Elementary school	1 (3.2)
High school	14 (45.2)
University or college	16 (51.6)
Missing values	2
Children	
Age (years), mean (SD)	9.4 (0.7)
Gender, girls, n (%)	14 (42.4)
BMI z-score, mean (SD)	
Baseline	2.9 (0.7)
4-year follow-up	2.5 (0.6)

SD: standard deviation. BMI: Body Mass Index. * Foreign background: parent and grandparents born outside Sweden or parent born in Sweden and grandparents born outside Sweden; missing value for 2 parents. ** Parents were classified with normal weight, overweight or obesity according to the international BMI criteria published by the World Health Organization [25].

## Data Availability

The data presented in this study are available on request from the corresponding author. The data are not publicly available due to privacy.
